# Bayesian Analysis of Three-Parameter Frechet Distribution with Medical Applications

**DOI:** 10.1155/2019/9089856

**Published:** 2019-03-12

**Authors:** Kamran Abbas, Nosheen Yousaf Abbasi, Amjad Ali, Sajjad Ahmad Khan, Sadaf Manzoor, Alamgir Khalil, Umair Khalil, Dost Muhammad Khan, Zamir Hussain, Muhammad Altaf

**Affiliations:** ^1^Department of Statistics, University of Azad Jammu and Kashmir, Muzaffarabad, Pakistan; ^2^Department of Statistics, Allama Iqbal Open University, Islamabad, Pakistan; ^3^Department of Statistics, Islamia College, Peshawar, Khyber Pakhtunkhwa, Pakistan; ^4^Department of Statistics, University of Peshawar, Khyber Pakhtunkhwa, Pakistan; ^5^Department of Statistics, Abdul Wali Khan University, Mardan, Khyber Pakhtunkhwa, Pakistan; ^6^Research Centre for Modeling and Simulation, National University of Sciences and Technology, Islamabad, Pakistan; ^7^Faculty of Basic Sciences and Humanities, University of Engineering and Technology, Taxila, Pakistan

## Abstract

The medical data are often filed for each patient in clinical studies in order to inform decision-making. Usually, medical data are generally skewed to the right, and skewed distributions can be the appropriate candidates in making inferences using Bayesian framework. Furthermore, the Bayesian estimators of skewed distribution can be used to tackle the problem of decision-making in medicine and health management under uncertainty. For medical diagnosis, physician can use the Bayesian estimators to quantify the effects of the evidence in increasing the probability that the patient has the particular disease considering the prior information. The present study focuses the development of Bayesian estimators for three-parameter Frechet distribution using noninformative prior and gamma prior under LINEX (linear exponential) and general entropy (GE) loss functions. Since the Bayesian estimators cannot be expressed in closed forms, approximate Bayesian estimates are discussed via Lindley's approximation. These results are compared with their maximum likelihood counterpart using Monte Carlo simulations. Our results indicate that Bayesian estimators under general entropy loss function with noninformative prior (BGENP) provide the smallest mean square error for all sample sizes and different values of parameters. Furthermore, a data set about the survival times of a group of patients suffering from head and neck cancer is analyzed for illustration purposes.

## 1. Introduction

Frechet distribution (FD) was introduced by Maurice Frechet (1878–1973) for largest extremes [1]. It had been derived with nonnegative initial variates. The FD deals with extreme events and also recognized as extreme value Type-II distribution. The cumulative distribution function of three-parameter FD is

(1) $$\begin{array}{c} F\left(x;\alpha ,\lambda ,\eta \right)=\exp\left[-{\left(\frac{x-\eta }{\lambda }\right)}^{-\alpha }\right],\;x>0,\alpha ,\lambda >0,\eta \in \left(-\infty ,\infty \right), \end{array}$$

where *α* is the shape, *λ* is the scale, and *η* is the location parameter. If *η*=0 then it becomes two-parameter FD. The corresponding probability density function is

(2) $$\begin{array}{c} f\left(x;\alpha ,\lambda ,\eta \right)=\frac{\alpha }{\lambda }{\left(\frac{x-\eta }{\lambda }\right)}^{-\left(\alpha +1\right)}\exp\left[-{\left(\frac{x-\eta }{\lambda }\right)}^{-\alpha }\right]. \end{array}$$

A number of authors have studied the estimation of its parameters, namely, Gumbel [2], Mann [3], Singh [4], and Hooda et al. [5]. Moreover, Afify [6] estimated the parameters of FD using principal components and least median of squares. Mubarak [7, 8] derived the best linear unbiased estimators and the best linear invariant estimators of location and scale parameters of FD under progressive Type-II censoring, respectively. Abbas and Tang [9] discussed classical as well as the Bayesian estimators of FD assuming that the shape parameter was known. Abbas and Tang [10] developed maximum likelihood and least squares estimators for FD with Type-II censored samples. Furthermore, Abbas and Tang [11, 12] derived the reference and matching priors for the Frechet stress-strength model and developed Bayesian estimators for FD under reference prior, respectively. Nasir and Aslam [13] obtained Bayes estimators of FD and their risks by using four loss functions under Gumbel Type-II prior and Levy prior. Yet, the Bayesian analysis of three-parameter FD is not conducted.

The aim of this paper is to develop Bayesian estimators for three-parameter FD using noninformative prior and gamma prior under two loss functions for the case of complete samples. Including this introduction section, the rest of the paper unfolds as follows: in Section 2, maximum likelihood estimators (MLEs) for the parameters are obtained. In Section 3, Bayesian estimators based on different loss functions by taking noninformative and gamma priors are derived. The proposed estimators are compared in terms of their mean squared error (MSE) in Section 4.  Section 5 illustrates the applications of proposed estimators using head and neck cancer data set. Finally, conclusions and recommendations are presented in Section 6.

## 2. Maximum Likelihood Estimation

Let *X*_1_, *X*_2_,…, *X*_*n*_ be random samples of size *n* from a three-parameter FD, then the likelihood function of (2) is

(3) $$\begin{array}{c} Ł\left(\underline{x};\alpha ,\lambda ,\eta \right)=\overset{n}{\underset{i=1}{\prod }}\left[\frac{\alpha }{\lambda }{\left(\frac{X-\eta }{\lambda }\right)}^{-\left(\alpha +1\right)}\exp\left[-{\left(\frac{X-\eta }{\lambda }\right)}^{-\alpha }\right]\right]. \end{array}$$

The corresponding log-likelihood function is

(4) $$\begin{array}{c} Ł=\text{log }Ł\left(\underline{x};\alpha ,\lambda ,\eta \right)=n\text{ log}\left(\alpha \right)+n\alpha \text{ log}\left(\lambda \right)-\left(\alpha +1\right)\overset{n}{\underset{i=1}{\sum }}\log\left(x_{i}-\eta \right)-{\overset{n}{\underset{i=1}{\sum }}\left[\frac{x_{i}-\eta }{\lambda }\right]}^{-\alpha }, \end{array}$$

from equation (4), we have

(5) $$\begin{array}{c} \frac{\partial Ł}{\partial \alpha }=\frac{n}{\alpha }+n\text{ log}\left(\lambda \right)-\overset{n}{\underset{i=1}{\sum }}\log\left(x_{i}-\eta \right)+\overset{n}{\underset{i=1}{\sum }}{\left[\frac{x_{i}-\eta }{\lambda }\right]}^{-\alpha }\times \log\left[\frac{x_{i}-\eta }{\lambda }\right]=0,\\ \frac{\partial Ł}{\partial \lambda }=\frac{n\alpha }{\lambda }-\frac{\alpha }{\lambda }\overset{n}{\underset{i=1}{\sum }}{\left[\frac{x_{i}-\eta }{\lambda }\right]}^{-\alpha }=0,\\ \frac{\partial Ł}{\partial \eta }=\left(\alpha +1\right)\overset{n}{\underset{i=1}{\sum }}{\left(x_{i}-\eta \right)}^{-1}-\frac{\alpha }{\lambda }\overset{n}{\underset{i=1}{\sum }}{\left[\frac{x_{i}-\eta }{\lambda }\right]}^{-\alpha -1}=0. \end{array}$$

Clearly, the above equations cannot be written in a closed form. Therefore, BFGS quasi-Newton optimization method (Broyden Fletcher GoldFarb Shanno, Battiti, and Masulli [14]) is applied to compute the MLEs.

## 3. Bayesian Estimation

In Bayesian estimation, we consider the two types of loss functions. The first is LINEX loss function, introduced by Varian [15]. This loss function was widely used by several authors, for example, Rojo [16], Basu and Ebrahimi [17], Pandey [18], Soliman [19], and Soliman et al. [20]. The second is general entropy loss function (GELF), defined by Calabria and Pulcini [21]. For Bayesian analysis, we need prior distribution. When prior information about the parameters is unavailable, then the noninformative prior can be considered for the Bayesian study. So, we supposed the noninformative form of priors for the all unknown parameters *α*, *λ*, and *η* of three-parameter FD as

(6) $$\begin{array}{c} \pi^{*}\left(\alpha ,\lambda ,\eta \right)\propto \left(\frac{1}{\alpha \lambda \eta }\right),\;\alpha ,\lambda ,\eta >0. \end{array}$$

If someone has a few information about parameters, then informative priors may be used for Bayesian analysis. It is noted that FD converts an inverse exponential distribution if its shape parameter equal to 1 and takes the form of the inverse Rayleigh distribution for shape parameter equal to 2, and when shape equal to 0.5, it approximates the inverse gamma distribution. So, we consider gamma prior for the scale parameter by assuming that shape parameter is known and independent priors for the shape and location parameters. Thus the proposed prior is

(7) $$\begin{array}{c} \pi_{1}\left(\alpha \right)\propto \frac{1}{\alpha },\;\alpha >0,\\ \pi_{2}\left(\eta \right)\propto \frac{1}{\eta },\;\eta >0,\\ \pi_{3}\left(\lambda \right)\propto \frac{b^{a}\lambda^{a-1}e^{-b\lambda }}{\Gamma \left(a\right)},\;\lambda >0,a,b>0. \end{array}$$

The joint prior distribution of parameters *α*, *λ*, and *η* is

(8) $$\begin{array}{c} \pi^{*}\left(\alpha ,\lambda ,\eta \right)\propto \frac{b^{a}\lambda^{a-1}e^{-b\lambda }}{\alpha \eta \Gamma \left(a\right)},\;\alpha ,\lambda ,\eta >0,a,b>0. \end{array}$$

The joint posterior density can be written as

(9) $$\begin{array}{c} \Phi^{*}\left(\alpha ,\lambda ,\eta \;|\;x\right)=\frac{L\left(\alpha ,\lambda ,\eta \;|\;x\right)\times \pi^{*}\left(\alpha ,\lambda ,\eta \right)}{\int_{\left(\alpha ,\lambda ,\eta \right)}L\left(\alpha ,\lambda ,\eta \;|\;x\right)\times \pi^{*}\left(\alpha ,\lambda ,\eta \right)\times d\alpha \;d\lambda \;d\eta }. \end{array}$$

Posterior distribution (9) takes a ratio form that cannot be reduced to a closed form. Therefore, we use Lindley's approximation [22] to get the Bayesian estimate, which can be written as

(10) $$\begin{array}{c} I\left(x\right)=u+\left(u_{1}b_{1}+u_{2}b_{2}+u_{3}b_{3}+b_{4}+b_{5}\right)+\frac{1}{2}\left[\Lambda_{1}\left(u_{1}\sigma_{11}+u_{2}\sigma_{12}+u_{3}\sigma_{13}\right)\right]+\frac{1}{2}\left(\Lambda_{2}\left(u_{1}\sigma_{21}+u_{2}\sigma_{22}+u_{3}\sigma_{23}\right)\right]+\frac{1}{2}\left(\Lambda_{3}\left(u_{1}\sigma_{31}+u_{2}\sigma_{32}+u_{3}\sigma_{33}\right)\right]. \end{array}$$

The detail of equation (10) is given in Appendix. Therefore, the approximate Bayesian estimators of parameters *α*, *λ*, and *η* by using noninformative prior under LINEX loss function are

(11) $$\begin{array}{c} {\hat{\alpha }}_{B_{L}\text{NP}}=\hat{\alpha }-\frac{1}{k_{2}}\log\left[1-k_{2}\left(-\frac{1}{\hat{\alpha }}\sigma_{11}-\frac{1}{\hat{\lambda }}\sigma_{12}-\frac{1}{\hat{\eta }}\sigma_{13}-\frac{k_{2}}{2}\sigma_{11}\right)+\frac{1}{2}\left(\Lambda_{1}\sigma_{11}+\Lambda_{2}\sigma_{21}+\Lambda_{3}\sigma_{31}\right)\right],\\ {\hat{\lambda }}_{B_{L}\text{NP}}=\hat{\lambda }-\frac{1}{k_{2}}\log\left[1-k_{2}\left(-\frac{1}{\hat{\alpha }}\sigma_{21}-\frac{1}{\hat{\lambda }}\sigma_{22}-\frac{1}{\hat{\eta }}\sigma_{23}-\frac{k_{2}}{2}\sigma_{22}\right)+\frac{1}{2}\left(\Lambda_{1}\sigma_{12}+\Lambda_{2}\sigma_{22}+\Lambda_{3}\sigma_{32}\right)\right],\\ {\hat{\eta }}_{B_{L}\text{NP}}=\hat{\eta }-\frac{1}{k_{2}}\log\left[1-k_{2}\left(-\frac{1}{\hat{\alpha }}\sigma_{31}-\frac{1}{\hat{\lambda }}\sigma_{32}-\frac{1}{\hat{\eta }}\sigma_{33}-\frac{k_{2}}{2}\sigma_{33}\right)+\frac{1}{2}\left(\Lambda_{1}\sigma_{13}+\Lambda_{2}\sigma_{23}+\Lambda_{3}\sigma_{33}\right)\right]. \end{array}$$

Bayesian estimators of parameters *α*, *λ*, and *η* with gamma prior under LINEX loss function are

(12) $$\begin{array}{c} {\hat{\alpha }}_{B_{L}\text{GP}}=\hat{\alpha }-\frac{1}{k_{2}}\log\left[1-k_{2}\left(-\frac{1}{\hat{\alpha }}\sigma_{11}+\left(\frac{a-1}{\hat{\lambda }}-b\right)\sigma_{12}-\frac{1}{\hat{\eta }}\sigma_{13}-\frac{k_{2}}{2}\sigma_{11}+\frac{1}{2}\left(\Lambda_{1}\sigma_{11}+\Lambda_{2}\sigma_{21}+\Lambda_{3}\sigma_{31}\right)\right)\right],\\ {\hat{\lambda }}_{B_{L}\text{GP}}=\hat{\lambda }-\frac{1}{k_{2}}\log\left[1-k_{2}\left(-\frac{1}{\hat{\alpha }}\sigma_{21}+\left(\frac{a-1}{\hat{\lambda }}-b\right)\sigma_{22}-\frac{1}{\hat{\eta }}\sigma_{23}-\frac{k_{2}}{2}\sigma_{22}+\frac{1}{2}\left(\Lambda_{1}\sigma_{12}+\Lambda_{2}\sigma_{22}+\Lambda_{3}\sigma_{32}\right)\right)\right],\\ {\hat{\eta }}_{B_{L}\text{GP}}=\hat{\eta }-\frac{1}{k_{2}}\log\left[1-k_{2}\left(-\frac{1}{\hat{\alpha }}\sigma_{31}+\left(\frac{a-1}{\hat{\lambda }}-b\right)\sigma_{32}-\frac{1}{\hat{\eta }}\sigma_{33}-\frac{k_{2}}{2}\sigma_{33}+\frac{1}{2}\left(\Lambda_{1}\sigma_{13}+\Lambda_{2}\sigma_{23}+\Lambda_{3}\sigma_{33}\right)\right)\right]. \end{array}$$

Similarly, Bayesian estimators of *α*, *λ*, and *η* using noninformative prior under GELF are

(13) $$\begin{array}{c} {\hat{\alpha }}_{{\text{BG}}_{E}\text{NP}}={\left[{\hat{\alpha }}^{-k_{2}}\left\{1-\frac{k_{2}}{\hat{\alpha }}\left(-\frac{1}{\hat{\alpha }}\sigma_{11}-\frac{1}{\hat{\lambda }}\sigma_{12}-\frac{1}{\hat{\eta }}\sigma_{13}-\frac{k_{2}+1}{2\hat{\alpha }}\sigma_{11}\right)+\frac{1}{2}\left(\Lambda_{1}\sigma_{11}+\Lambda_{2}\sigma_{21}+\Lambda_{3}\sigma_{31}\right)\right\}\right]}^{-\left(1/k_{2}\right)},\\ {\hat{\lambda }}_{{\text{BG}}_{E}\text{NP}}={\left[{\hat{\lambda }}^{-k_{2}}\left\{1-\frac{k_{2}}{\hat{\lambda }}\left(-\frac{1}{\hat{\alpha }}\sigma_{21}-\frac{1}{\hat{\lambda }}\sigma_{22}-\frac{1}{\hat{\eta }}\sigma_{23}-\frac{k_{2}+1}{2\hat{\lambda }}\sigma_{22}\right)+\frac{1}{2}\left(\Lambda_{1}\sigma_{12}+\Lambda_{2}\delta_{22}+\Lambda_{3}\sigma_{32}\right)\right\}\right]}^{-\left(1/k_{2}\right)},\\ {\hat{\eta }}_{{\text{BG}}_{E}\text{NP}}={\left[{\hat{\eta }}^{-k_{2}}\left\{1-\frac{k_{2}}{\hat{\eta }}\left(-\frac{1}{\hat{\alpha }}\sigma_{31}-\frac{1}{\hat{\lambda }}\sigma_{32}-\frac{1}{\hat{\eta }}\sigma_{33}-\frac{k_{2}+1}{2\hat{\eta }}\sigma_{33}\right)+\frac{1}{2}\left(\Lambda_{1}\sigma_{13}+\Lambda_{2}\delta_{23}+\Lambda_{3}\sigma_{33}\right)\right\}\right]}^{-\left(1/k_{2}\right)}. \end{array}$$

Bayesian estimators of parameters *α*, *λ*, and *η* with gamma prior under GELF are

(14) $$\begin{array}{c} {\hat{\alpha }}_{{\text{BG}}_{E}\text{GP}}={\left[{\hat{\alpha }}^{-k_{2}}\left\{1-\frac{k_{2}}{\hat{\alpha }}\left(-\frac{1}{\hat{\alpha }}\sigma_{11}+\left(\frac{a-1}{\hat{\lambda }}-b\right)\sigma_{12}-\frac{1}{\hat{\eta }}\sigma_{13}-\frac{k_{2}+1}{2\hat{\alpha }}\sigma_{11}\right)+\frac{1}{2}\left(\Lambda_{1}\sigma_{11}+\Lambda_{2}\sigma_{21}+\Lambda_{3}\sigma_{31}\right)\right\}\right]}^{-\left(1/k_{2}\right)},\\ {\hat{\lambda }}_{{\text{BG}}_{E}\text{GP}}={\left[{\hat{\lambda }}^{-k_{2}}\left\{1-\frac{k_{2}}{\hat{\lambda }}\left(-\frac{1}{\hat{\alpha }}\sigma_{21}+\left(\frac{a-1}{\hat{\lambda }}-b\right)\sigma_{22}-\frac{1}{\hat{\eta }}\sigma_{23}-\frac{k_{2}+1}{2\hat{\lambda }}\sigma_{22}\right)+\frac{1}{2}\left(\Lambda_{1}\sigma_{12}+\Lambda_{2}\delta_{22}+\Lambda_{3}\sigma_{32}\right)\right\}\right]}^{-\left(1/k_{2}\right)},\\ {\hat{\eta }}_{{\text{BG}}_{E}\text{GP}}={\left[{\hat{\eta }}^{-k_{2}}\left\{1-\frac{k_{2}}{\hat{\eta }}\left(-\frac{1}{\hat{\alpha }}\sigma_{31}+\left(\frac{a-1}{\hat{\lambda }}-b\right)\sigma_{32}-\frac{1}{\hat{\eta }}\sigma_{33}-\frac{k_{2}+1}{2\hat{\eta }}\sigma_{33}\right)+\frac{1}{2}\left(\Lambda_{1}\sigma_{13}+\Lambda_{2}\delta_{23}+\Lambda_{3}\sigma_{33}\right)\right\}\right]}^{-\left(1/k_{2}\right)}, \end{array}$$

where $\hat{\alpha }$, $\hat{\lambda }$, and $\hat{\eta }$ are the ML estimates of parameters *α*, *λ*, and *η*, respectively. Further, the observed Fisher information matrix is obtained by taking the second and mixed partial derivatives of equation (4) with respect to parameters *α*, *λ*, and *η*, respectively, provided in Appendix.

## 4. Simulation Study

To demonstrate the performance of the proposed Bayesian estimators with their ML counterpart in terms of biases and MSE (within parenthesis), different sample sizes and different values of parameters are considered using Monte Carlo simulation. Monte Carlo simulation is conducted as follows:

1. Take the initial values of *α*, *λ*, and *η*, respectively. Samples are generated from the FD using inverse transformation technique, i.e., *X*(*F*)=*η*+*λ*(−ln *U*)^−(1/*α*)^, where *U* is uniformly distributed random variable over the interval of [0, 1] and considering *x* > *η*.
2. Calculate the ML and Bayesian estimators of *g*(*α*, *λ*, *η*) by 1/*R*∑_*i*=1_^*R*^ *g*(*α*_*i*_, *λ*_*i*_, *η*_*i*_), where *g*(*α*_*i*_, *λ*_*i*_, *η*_*i*_) is the function of *α*, *λ*, and *η* using informative and noninformative priors and *R* is the number of iterations.
3. The process is replicated 3000 times for each sample size and averages of these estimates and the corresponding MSEs (within parenthesis) were calculated for each method.

The results are listed in Tables 1 [Table tab2] [Table tab3]–4 for comparison purposes.  Table 1 contains simulation results for the case where *α*=1, *λ*=2, and *η*=3, and Table 2 presents the simulation results when *α*=1, *λ*=1, and *η*=4. Moreover, Tables 3 and 4 comprise the results for the case where *α*=0.5, *λ*=1.5, and *η*=3 and *α*=1, *λ*=2, and *η*=4, respectively. From the results of the simulation study, conclusions are drawn regarding the behavior of the estimators, which are summarized below:

- MSE decreases for both ML and Bayesian method when the sample sizes increases.
- In terms of MSE, the BGENP estimator provides the smallest MSE for all samples sizes and different values of parameters.
- Apparently, Bayesian and MLEs become better when the sample size increases. However, similar performance can be observed for large sample sizes.
- Based on simulation study and real data analysis, we suggest that the BGENP estimators in each scenario execute considerably, because the MSE is significantly smaller.

## 5. Data Analysis

For exemplification purposes, the data set presented in Table 5 reported by Efron [23] represents the survival times of a group of patients suffering from head and neck cancer and treated using a combination of radiotherapy and chemotherapy. The data set consists of 44 observations.

**Figure 1:**
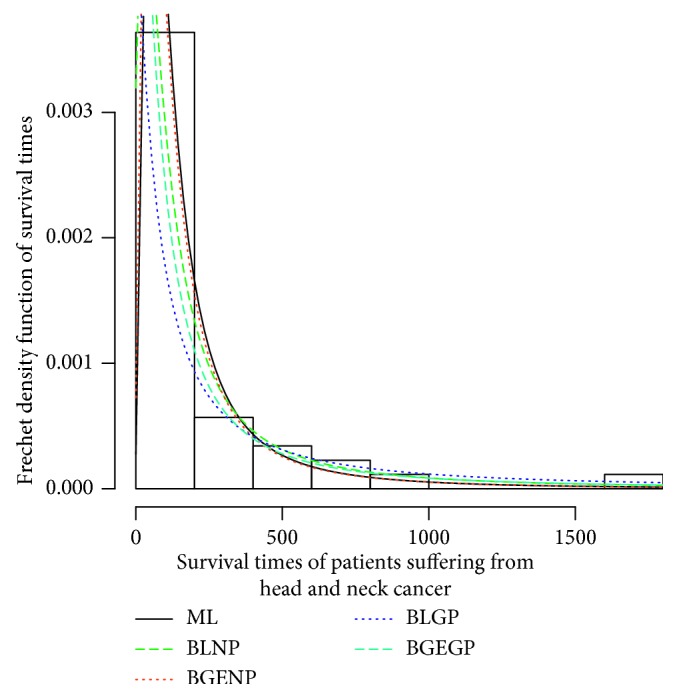
Comparison of estimation methods.

Parametric analysis is performed to determine the best-fitted probability distribution function that characterizes the survival times of a group of patients suffering from head and neck cancer. The distribution in Figure 1 is highly skewed to the right. The distribution curve is asymmetric being stretched out to the right. Among the skewed distributions, FD is fitted to survival times of a group of patients suffering from head and neck cancer, parameters are estimated by using ML and Bayesian methods, and the results are presented in Table 6 for comparison purposes. The Kolmogorov–Smirnov (KS) test along with *P* values is used to quantify the model. Further, *P* values of KS test are significant at 5% level of significance except BLGP, which may indicate that the rest of the estimators are most appropriate for estimating the parameter of FD. Moreover, different estimates can also be visualized in Figure 1, in which the *x*-axis represents the survival times of a group of patients suffering from head and neck cancer while the Frechet density function of survival is taken on the *y*-axis.

## 6. Conclusion and Recommendations

Statistical decision theory addresses the state of uncertainty and provides a rational framework for dealing problems of medical decision-making. The Bayesian paradigm represents the probabilistic relationships between diseases and symptoms. Although medical data are generally skewed to the right, positively skewed distributions are reasonably competitive when describing unimodal medical data. In this study, an attempt has been made to examine the Bayesian estimators for three-parameter FD with medical applications. The Bayesian estimators are obtained using LINEX and GE loss functions considering gamma and noninformative priors through Lindley approximation. It is concluded that BGENP performed quite well in estimating the parameters of FD in terms of MSE as compared to other estimators. However, Bayesian and MLEs get closer in terms of MSEs for larger sample sizes. Though computation of Lindley's method is based on the MLEs, it performs well for various sample sizes. The speed of convergence of Lindley's method is so fast for all problems and may rely on initial points. Based on these findings, it can be safely concluded that parametric FD is most suitable for describing the medical related data.

The study will offer a framework for testing features of other skewed distributions considering Bayesian framework with medical data. Thus, providing a more detailed and accurate understanding of the distribution of medical data and professionals can make decisions on rational bases. Moreover, the problem of Bayes estimation for three-parameter FD can be extended to include informative priors and also to consider other techniques such as MCMC (Markov Chain Monte Carlo) methods, and Laplace approximation can be used to get the posterior summaries, investigate their performances, and compare them with those of the MLEs.

## Figures and Tables

**Table 1:** Average estimates for *α*=1, *λ*=2, and *η*=3 and MSEs (within parenthesis).

*n*	Methods	*α*=1	*λ*=2	*η*=3
25	ML	1.0846 (0.2084)	2.1346 (1.0679)	2.9279 (0.4305)
	BLNP	0.9628 (0.1912)	1.9780 (1.0348)	2.8178 (0.4774)
	BGENP	1.0412 (0.1562)	1.9997 (0.8626)	2.8577 (0.4294)
	BLGP	0.9616 (0.1911)	1.9777 (1.0345)	2.8172 (0.4779)
	BGEGP	0.9589 (0.1768)	1.8765 (0.9230)	2.7209 (0.4913)

50	ML	1.0066 (0.0455)	1.9707 (0.2594)	3.0378 (0.0710)
	BLNP	0.9054 (0.0514)	1.8329 (0.2807)	2.9466 (0.0768)
	BGENP	0.9818 (0.0423)	1.8764 (0.2473)	2.9920 (0.0628)
	BLGP	0.9042 (0.0516)	1.8328 (0.2807)	2.9459 (0.0770)
	BGEGP	0.9080 (0.0452)	1.7590 (0.2697)	2.8678 (0.0927)

70	ML	0.9956 (0.0269)	1.9795 (0.1822)	3.0394 (0.0569)
	BLNP	0.9035 (0.0344)	1.8503 (0.2008)	2.9569 (0.0605)
	BGENP	0.9763 (0.0258)	1.8987 (0.1769)	3.0038 (0.0474)
	BLGP	0.9023 (0.0346)	1.8500 (0.2008)	2.9561 (0.0606)
	BGEGP	0.9063 (0.0309)	1.7827 (0.2000)	2.8909 (0.0717)

100	ML	1.0127 (0.0212)	2.0353 (0.1197)	3.0051 (0.0386)
	BLNP	0.9296 (0.0246)	1.9157 (0.1235)	2.9306 (0.0456)
	BGENP	0.9975 (0.0201)	1.9672 (0.1116)	2.9778 (0.0302)
	BLGP	0.9284 (0.0248)	1.9154 (0.1235)	2.9299 (0.0457)
	BGEGP	0.9307 (0.0225)	1.8522 (0.1236)	2.8773 (0.0568)

MLE: maximum likelihood estimator; BLNP: Bayesian estimator under LINEX loss function with noninformative prior; BGENP: Bayesian estimator under general entropy loss function with noninformative prior; BLGP: Bayesian estimator under LINEX loss function with gamma prior; BGEGP: Bayesian estimator under general entropy loss function with gamma prior.

**Table 2 tab2:** Average estimates for *α*=1, *λ*=1, and *η*=4 and MSEs (within parenthesis).

*n*	Methods	*α*=1	*λ*=1	*η*=4
25	ML	1.0686 (0.2168)	1.0192 (0.3295)	3.9716 (0.1756)
	BLNP	0.9475 (0.2033)	0.8892 (0.3356)	3.8863 (0.1969)
	BGENP	1.0262 (0.1669)	0.9649 (0.2828)	3.9189 (0.1682)
	BLGP	0.9477 (0.2030)	0.8893 (0.3355)	3.8864 (0.1971)
	BGEGP	0.94626 (0.1859)	0.8966 (0.3014)	3.7904 (0.2299)

50	ML	1.0005 (0.0434)	1.0020 (0.0799)	4.0227 (0.0185)
	BLNP	0.8994 (0.0507)	0.8890 (0.0888)	3.9533 (0.0222)
	BGENP	0.9759 (0.0406)	0.9620 (0.0749)	3.9905 (0.0180)
	BLGP	0.8994 (0.0506)	0.8890 (0.0887)	3.9533 (0.0222)
	BGEGP	0.9036 (0.0444)	0.8946 (0.0765)	3.8876 (0.0360)

70	ML	1.0070 (0.0294)	0.9931 (0.0457)	4.0136 (0.0149)
	BLNP	0.9147 (0.0347)	0.8897 (0.0564)	3.9524 (0.0184)
	BGENP	0.9873 (0.0277)	0.9606 (0.0444)	3.9897 (0.0147)
	BLGP	0.9147 (0.0346)	0.8897 (0.0564)	3.9525 (0.0184)
	BGEGP	0.9179 (0.0310)	0.8952 (0.0495)	3.9023 (0.0277)

100	ML	0.9807 (0.0112)	0.9672 (0.0335)	4.0253 (0.0078)
	BLNP	0.8987 (0.0204)	0.8735 (0.0470)	3.9735 (0.0086)
	BGENP	0.9664 (0.0110)	0.9414 (0.0330)	4.0084 (0.0076)
	BLGP	0.8988 (0.0203)	0.8735 (0.0470)	3.9735 (0.0086)
	BGEGP	0.9027 (0.0186)	0.8795 (0.0420)	3.9383 (0.0128)

**Table 3 tab3:** Average estimates for *α*=0.5, *λ*=1.5, and *η*=3 and MSEs (within parenthesis).

*n*	Methods	*α*=0.5	*λ*=1.5	*η*=3
25	ML	0.4943 (0.0169)	1.3949 (0.5350)	3.0601 (0.0211)
	BLNP	0.3977 (0.0256)	1.2209 (0.5768)	2.9876 (0.0212)
	BGENP	0.4804 (0.0159)	1.2765 (0.4703)	3.0285 (0.0210)
	BLNP	0.3966 (0.0259)	1.2207 (0.5766)	2.9868 (0.0212)
	BGEGP	0.4379 (0.0171)	1.1824 (0.4775)	2.9268 (0.0310)

50	ML	0.4937 (0.0058)	1.5372 (0.2661)	3.0213 (0.0063)
	BLNP	0.4137 (0.0127)	1.3767 (0.2674)	2.9607 (0.0088)
	BGENP	0.4849 (0.0057)	1.4323 (0.2274)	3.0012 (0.0062)
	BLGP	0.4122 (0.0130)	1.3765 (0.2674)	2.9595 (0.0089)
	BGEGP	0.4450 (0.0077)	1.3285 (0.2282)	2.9166 (0.0161)

70	ML	0.4946 (0.0033)	1.4658 (0.1306)	3.0220 (0.0033)
	BLNP	0.4232 (0.0089)	1.3162 (0.1576)	2.9696 (0.0044)
	BGENP	0.4877 (0.0030)	1.3800 (0.1264)	3.0073 (0.0031)
	BLGP	0.4220 (0.0091)	1.3160 (0.1575)	2.9686 (0.0045)
	BGEGP	0.4500 (0.0053)	1.2795 (0.1485)	2.9364 (0.0084)

100	ML	0.5030 (0.0025)	1.4499 (0.1150)	3.0166 (0.0018)
	BLNP	0.4401 (0.0059)	1.3114 (0.1424)	2.9719 (0.0027)
	BGENP	0.4977 (0.0024)	1.3777 (0.1136)	3.0060 (0.0017)
	BLGP	0.4389 (0.0060)	1.3113 (0.1423)	2.9711 (0.0028)
	BGEGP	0.4621 (0.0036)	1.2789 (0.1366)	2.9477 (0.0052)

**Table 4:** Average estimates for *α*=1, *λ*=2, and *η*=4 and MSEs (within parenthesis).

*n*	Methods	*α*=1	*λ*=2	*η*=4
25	ML	1.0071 (0.2034)	1.9144 (0.9570)	4.0738 (0.4348)
	BLNP	0.8881 (0.2050)	1.7585 (0.9943)	3.9681 (0.4455)
	BGENP	0.9680 (0.1688)	1.7917 (0.8742)	3.9883 (0.4324)
	BLGP	0.8870 (0.2052)	1.7583 (0.9942)	3.9675 (0.4457)
	BGEGP	0.8908 (0.1781)	1.6776 (0.8783)	3.8290 (0.4487)

50	ML	0.9946 (0.0536)	1.9231 (0.2714)	4.0420 (0.0857)
	BLNP	0.8938 (0.0613)	1.7856 (0.3072)	3.9522 (0.0916)
	BGENP	0.9701 (0.0503)	1.8314 (0.2703)	3.9861 (0.0854)
	BLGP	0.8927 (0.0615)	1.7853 (0.3072)	3.9515 (0.0918)
	BGEGP	0.8971 (0.0538)	1.7163 (0.3013)	3.8479 (0.1183)

70	ML	0.9767 (0.0263)	1.9316 (0.2179)	4.0576 (0.0562)
	BLNP	0.8854 (0.0371)	1.8029 (0.2470)	3.9761 (0.0567)
	BGENP	0.9580 (0.0259)	1.8523 (0.2161)	4.0141 (0.0558)
	BLGP	0.8842 (0.0374)	1.8029 (0.2470)	3.9754 (0.0567)
	BGEGP	0.8893 (0.0335)	1.7384 (0.2461)	3.8896 (0.0716)

100	ML	1.0186 (0.0240)	2.0271 (0.1457)	3.9930 (0.0470)
	BLNP	0.9354 (0.0263)	1.9079 (0.1512)	3.9188 (0.0563)
	BGENP	1.0034 (0.0225)	1.9596 (0.1372)	3.9593 (0.0405)
	BLGP	0.9341 (0.0264)	1.9076 (0.1512)	3.9181 (0.0564)
	BGEGP	0.9362 (0.0240)	1.8451 (0.1490)	3.8493 (0.0752)

**Table 5:** Survival times of a group of patients suffering from head and neck cancer.

12.20	23.56	23.74	25.87	31.98	37	41.35	47.38	55.46	58.36	63.47
68.46	78.26	74.47	81.43	84	92	94	110	112	119	127
130	133	140	146	155	159	173	179	194	195	209
249	281	319	339	432	469	519	633	725	817	1776

**Table 6:** Goodness of fit test.

Methods	Estimators			KS test	
	*α*	*λ*	*η*	*D*	*P* value
ML	1.5292	120.7537	−33.62417	0.0553	0.9982
BLNP	1.1071	519.9316	−37.7096	0.1098	0.6236
BGENP	1.4870	114.8435	−35.1019	0.0843	0.8865
BLGP	0.6747	114.6567	−39.13914	0.2043	0.0435
BGEGP	0.9497	93.2270	−35.9787	0.1936	0.0641

## Data Availability

Our work is mainly a methodological development and has been applied on secondary data, but if required, data will be provided.

## Appendix

The observed Fisher information matrix can be written as

(A.1) $$\begin{array}{c} I\left(\alpha ,\lambda ,\eta \right)=\left(\begin{array}{ccc} -\frac{\partial^{2}Ł}{\partial \alpha^{2}} & -\frac{\partial^{2}Ł}{\partial \alpha \lambda } & -\frac{\partial^{2}Ł}{\partial \alpha \eta }\\ -\frac{\partial^{2}Ł}{\partial \lambda \alpha } & -\frac{\partial^{2}Ł}{\partial \lambda^{2}} & -\frac{\partial^{2}Ł}{\partial \lambda \eta }\\ -\frac{\partial^{2}Ł}{\partial \eta \alpha } & -\frac{\partial^{2}Ł}{\partial \eta \lambda } & -\frac{\partial^{2}Ł}{\partial \eta^{2}} \end{array}\right), \end{array}$$

where

(A.2) $$\begin{array}{c} \frac{\partial^{2}L}{\partial \alpha^{2}}=-\frac{n}{\alpha^{2}}-\overset{n}{\underset{i=1}{\sum }}{\left[\frac{x_{i}-\eta }{\lambda }\right]}^{-\alpha }\log^{2}\left[\frac{x_{i}-\eta }{\lambda }\right],\\ \frac{\partial^{2}L}{\partial \alpha \;\lambda }=\frac{n}{\lambda }+\frac{\alpha }{\lambda }\overset{n}{\underset{i=i}{\sum }}{\left[\frac{x_{i}-\eta }{\lambda }\right]}^{-\alpha }\log\left[\frac{x_{i}-\eta }{\lambda }\right]-\frac{1}{\lambda }\overset{n}{\underset{i=1}{\sum }}{\left[\frac{x_{i}-\eta }{\lambda }\right]}^{-\alpha },\\ \frac{\partial^{2}L}{\partial \alpha \eta }=\overset{n}{\underset{i=1}{\sum }}\left[\frac{1}{x_{i}-\eta }\right]+\frac{\alpha }{\lambda }\overset{n}{\underset{i=1}{\sum }}{\left[\frac{x_{i}-\eta }{\lambda }\right]}^{-\alpha -1}\log\left[\frac{x_{i}-\eta }{\lambda }\right]-\overset{n}{\underset{i=1}{\sum }}{\left[\frac{x_{i}-\eta }{\lambda }\right]}^{-\alpha }{\left\{x_{i}-\eta \right\}}^{-1},\\ \frac{\partial^{2}L}{\partial \lambda \alpha }=\frac{n}{\lambda }+\frac{\alpha }{\lambda }\overset{n}{\underset{i=i}{\sum }}{\left[\frac{x_{i}-\eta }{\lambda }\right]}^{-\alpha }\log\left[\frac{x_{i}-\eta }{\lambda }\right]-\frac{1}{\lambda }\overset{n}{\underset{i=1}{\sum }}{\left[\frac{x_{i}-\eta }{\lambda }\right]}^{-\alpha },\\ \frac{\partial^{2}L}{\partial \lambda^{2}}=\frac{n\alpha }{\lambda }-\frac{\alpha \left(\alpha -1\right)}{\lambda^{2}}\overset{n}{\underset{i=1}{\sum }}{\left[\frac{x_{i}-\eta }{\lambda }\right]}^{-\alpha },\\ \frac{\partial^{2}L}{\partial \lambda \eta }=-\frac{\alpha^{2}}{\lambda }\overset{n}{\underset{i=1}{\sum }}{\left[\frac{x_{i}-\eta }{\lambda }\right]}^{-\left(\alpha +1\right)},\\ \frac{\partial^{2}L}{\partial \eta \alpha }=\overset{n}{\underset{i=1}{\sum }}\left[\frac{1}{x_{i}-\eta }\right]+\frac{\alpha }{\lambda }\overset{n}{\underset{i=1}{\sum }}{\left[\frac{x_{i}-\eta }{\lambda }\right]}^{-\alpha -1}\log\left[\frac{x_{i}-\eta }{\lambda }\right]-\frac{1}{\lambda }\overset{n}{\underset{i=1}{\sum }}{\left[\frac{x_{i}-\eta }{\lambda }\right]}^{-\alpha -1},\\ \frac{\partial^{2}L}{\partial \eta \lambda }=-\frac{\alpha^{2}}{\lambda }\overset{n}{\underset{i=1}{\sum }}{\left[\frac{x_{i}-\eta }{\lambda }\right]}^{-\left(\alpha +1\right)},\\ \frac{\partial^{2}L}{\partial \eta^{2}}=\left(\alpha +1\right)\overset{n}{\underset{i=1}{\sum }}\left[\frac{2}{x_{i}-\eta }\right]-\frac{\alpha \left(\alpha -1\right)}{\lambda^{2}}\overset{n}{\underset{i=1}{\sum }}{\left[\frac{x_{i}-\eta }{\lambda }\right]}^{-\alpha -1}. \end{array}$$

From equation (10), we have

(A.3) $$\begin{array}{c} \Lambda_{1}=\sigma_{11}L_{\alpha \alpha \alpha }+2\sigma_{12}L_{\alpha \lambda \alpha }+2\sigma_{13}L_{\alpha \eta \alpha }+2\sigma_{23}L_{\lambda \eta \alpha }+\sigma_{22}L_{\lambda \lambda \alpha }+\sigma_{33}L_{\eta \eta \alpha },\\ \Lambda_{2}=\sigma_{11}L_{\alpha \alpha \lambda }+2\sigma_{12}L_{\alpha \lambda \lambda }+2\sigma_{13}L_{\alpha \eta \lambda }+2\sigma_{23}L_{\lambda \eta \lambda }+\sigma_{22}L_{\lambda \lambda \lambda }+\sigma_{33}L_{\eta \eta \lambda },\\ \Lambda_{3}=\sigma_{11}L_{\alpha \alpha \eta }+2\sigma_{12}L_{\alpha \lambda \eta }+2\sigma_{13}L_{\alpha \eta \eta }+2\sigma_{23}L_{\lambda \eta \eta }+\sigma_{22}L_{\lambda \lambda \eta }+\sigma_{33}L_{\eta \eta \eta }, \end{array}$$

and for *i*=1,2,3,

(A.4) $$\begin{array}{c} b_{i}=\tau_{1}\sigma_{i1}+\tau_{2}\sigma_{i2}+\tau_{3}\sigma_{i3},\\ b_{4}=u_{12}\sigma_{12}+u_{13}\sigma_{13}+u_{23}\sigma_{23},\\ b_{5}=\frac{1}{2}\left(u_{11}\sigma_{11}+u_{22}\sigma_{22}+u_{33}\sigma_{33}\right),\\ \tau_{i}=\frac{\partial \tau }{\partial \phi_{i}},\;i=1,2,\\ u_{i}=\frac{\partial u\left(\phi_{1},\phi_{2},\phi_{3}\right)}{\partial \phi_{i}},\;i=1,2,\\ u_{ij}=\frac{\partial u^{2}\left(\phi_{1},\phi_{2},\phi_{3}\right)}{\partial \phi_{i},\phi_{j}},\;i,j=1,2,\\ {Ł}_{ij}=\frac{\partial u^{2}\left(\phi_{1},\phi_{2},\phi_{3}\right)}{\partial \phi_{i}\partial \phi_{j}},\;i,j=1,2,\\ {Ł}_{ijk}=\frac{\partial u^{3}\left(\phi_{1},\phi_{2},\phi_{3}\right)}{\partial \phi_{i}\partial \phi_{j}\partial \phi_{k}},\;i,j,k=1,2,3,\\ \sigma_{ij}=-\frac{1}{{Ł}_{ij}}. \end{array}$$

Moreover, *u*(*ϕ*_1_, *ϕ*_2_, *ϕ*_3_) represents the first, second, and third parameter, respectively. *τ*_*i*_ denotes the derivatives of log of prior w.r.t. *ϕ*_1_, *ϕ*_2_, and *ϕ*_3_. *Ł*_*ij*_ inverse of Fisher information and *Ł*_*ijk*_ are third derivatives obtained from equation (4).

The values of *L*_*ijk*_ used in Lindley's approximation are given as

(A.5) $$\begin{array}{c} {Ł}_{\alpha \alpha \alpha }=\frac{2n}{\alpha^{3}}+\overset{n}{\underset{i=1}{\sum }}{\left[\frac{x_{i}-\eta }{\lambda }\right]}^{-\alpha }\times \;\;\log^{3}\left[\frac{x_{i}-\eta }{\lambda }\right],\\ {Ł}_{\lambda \lambda \lambda }=\frac{2n\alpha }{\lambda^{3}}-\frac{\alpha \left(\alpha -1\right)\left(\alpha -2\right)}{\lambda^{3}}\overset{n}{\underset{i=1}{\sum }}{\left[\frac{x_{i}-\eta }{\lambda }\right]}^{-\alpha },\\ {Ł}_{\eta \eta \eta }=2\left(\alpha +1\right)\overset{n}{\underset{i=1}{\sum }}\left[\frac{1}{x_{i}-\eta }\right]-\frac{\alpha \left(\alpha +1\right)\left(\alpha +2\right)}{\lambda^{3}}{\overset{n}{\underset{i=1}{\sum }}\frac{x_{i}-\eta }{\lambda }}^{-\left(\alpha +3\right)},\\ {Ł}_{\lambda \eta \eta }={Ł}_{\eta \lambda \eta }={Ł}_{\eta \eta \lambda }=-\frac{\alpha^{2}\left(\alpha +1\right)}{\delta^{3}}\overset{n}{\underset{i=1}{\sum }}{\left[\frac{x_{i}-\lambda }{\lambda }\right]}^{-\left(\alpha +2\right)},\\ {Ł}_{\lambda \lambda \eta }={Ł}_{\lambda \eta \lambda }={Ł}_{\eta \lambda \lambda }=-\frac{\alpha^{2}\left(\alpha -1\right)}{\lambda^{3}}\overset{n}{\underset{i=1}{\sum }}{\left[\frac{x_{i}-\eta }{\lambda }\right]}^{-\left(\alpha +1\right)},\\ {Ł}_{\alpha \alpha \lambda }=\frac{2}{\lambda }\overset{n}{\underset{i=i}{\sum }}{\left[\frac{x_{i}-\eta }{\lambda }\right]}^{-\alpha }\times \text{ log}\left[\frac{x_{i}-\eta }{\lambda }\right]-\frac{\alpha }{\lambda }\overset{n}{\underset{i=1}{\sum }}{\left[\frac{x_{i}-\eta }{\lambda }\right]}^{-\alpha }\times \;\;\log^{2}\left[\frac{x_{i}-\eta }{\lambda }\right]\\ ={Ł}_{\alpha \lambda \alpha }={Ł}_{\lambda \alpha \alpha },\\ {Ł}_{\alpha \eta \lambda }=\frac{\alpha^{2}}{\lambda^{2}}\overset{n}{\underset{i=1}{\sum }}{\left[\frac{x_{i}-\eta }{\lambda }\right]}^{-\left(\alpha +1\right)}\times \text{ log}\left[\frac{x_{i}-\eta }{\lambda }\right]-\frac{2\alpha }{\lambda^{2}}\overset{n}{\underset{i=1}{\sum }}{\left[\frac{x_{i}-\eta }{\lambda }\right]}^{-\left(\alpha +1\right)}\\ ={Ł}_{\eta \alpha \lambda }={Ł}_{\lambda \alpha \eta }={Ł}_{\alpha \lambda \eta }={Ł}_{\lambda \eta \alpha }={Ł}_{\eta \lambda \alpha },\\ {Ł}_{\alpha \alpha \eta }={Ł}_{\alpha \eta \alpha }={Ł}_{\eta \alpha \alpha }=-\frac{\alpha }{\lambda }\overset{n}{\underset{i=1}{\sum }}{\left[\frac{x_{i}-\eta }{\lambda }\right]}^{-\left(\alpha +1\right)}\times \;\;\log^{2}\left[\frac{x_{i}-\eta }{\lambda }\right]+\frac{2}{\lambda }\overset{n}{\underset{i=1}{\sum }}{\left[\frac{x_{i}-\eta }{\lambda }\right]}^{-\alpha -1}\times \text{ log}\left[\frac{x_{i}-\eta }{\lambda }\right],\\ {Ł}_{\alpha \lambda \lambda }={Ł}_{\lambda \alpha \lambda }={Ł}_{\lambda \lambda \alpha }=-\frac{n}{\lambda^{2}}+\frac{\alpha^{2}}{\lambda^{2}}\overset{n}{\underset{i=1}{\sum }}{\left[\frac{x_{i}-\eta }{\lambda }\right]}^{-\alpha }\log\left[\frac{x_{i}-\eta }{\lambda }\right]-\frac{\alpha }{\lambda^{2}}\overset{n}{\underset{i=1}{\sum }}{\left[\frac{x_{i}-\eta }{\lambda }\right]}^{-\alpha }\times \text{ log}\left[\frac{x_{i}-\eta }{\lambda }\right]-\frac{2\alpha }{\lambda^{2}}\overset{n}{\underset{i=1}{\sum }}{\left[\frac{x_{i}-\eta }{\lambda }\right]}^{-\alpha }+\frac{1}{\lambda^{2}}\overset{n}{\underset{i=1}{\sum }}{\left[\frac{x_{i}-\eta }{\lambda }\right]}^{-\alpha },\\ {Ł}_{\alpha \eta \eta }={Ł}_{\eta \alpha \eta }={Ł}_{\eta \eta \alpha }=\overset{n}{\underset{i=1}{\sum }}{\left(x_{i}-\eta \right)}^{-2}+\frac{\alpha^{2}}{\lambda^{2}}\overset{n}{\underset{i=1}{\sum }}{\left[\frac{x_{i}-\eta }{\lambda }\right]}^{-\left(\alpha +2\right)}\log\left[\frac{x_{i}-\eta }{\lambda }\right]+\frac{\alpha }{\lambda^{2}}\overset{n}{\underset{i=1}{\sum }}{\left[\frac{x_{i}-\eta }{\lambda }\right]}^{-\left(\alpha +2\right)}\log\left[\frac{x_{i}-\eta }{\lambda }\right]-\frac{1}{\lambda^{2}}\overset{n}{\underset{i=1}{\sum }}{\left[\frac{x_{i}-\eta }{\lambda }\right]}^{-\left(\alpha +2\right)}-\frac{2\alpha }{\lambda^{2}}\overset{n}{\underset{i=1}{\sum }}{\left[\frac{x_{i}-\eta }{\lambda }\right]}^{-\left(\alpha +2\right)}. \end{array}$$
